# Can a pharmacy intervention improve the metabolic risks of mental health patients? Evaluation of a novel collaborative service

**DOI:** 10.1186/s12913-016-1406-6

**Published:** 2016-04-26

**Authors:** Husna Maulavizada, Lynne Emmerton, Hendrika Laetitia Hattingh

**Affiliations:** School of Pharmacy, Curtin University, GPO Box U1987, Perth, WA 6845 Australia

**Keywords:** Community pharmacy, pharmacist, Nurse practitioner, Mental health, Metabolic risk

## Abstract

**Background:**

The pressure on healthcare services worldwide has driven the incorporation of disease state management services within community pharmacies in developed countries. Pharmacists are recognised as the most accessible healthcare professionals, and the incorporation of these services facilitates patient care. In Australia, the opportunity to manage pharmacy patients with mental illness has been underutilised, despite the existence of service models for other chronic conditions. This paper is an independent evaluation of a novel service developed by a community pharmacy in Perth, Western Australia. The service represents collaboration between a nurse practitioner and community pharmacy staff in the management of mental health patients with metabolic risks.

**Methods:**

We applied practice service standards for Australian community pharmacies to develop an evaluation framework for this novel service. This was followed by semi-structured interviews with staff members at the study pharmacy to explore service processes and procedures. Descriptive analysis of interviews was supplemented with analysis of patients’ biometric data. All data were evaluated against the developed framework.

**Results:**

The evaluation framework comprised 13 process, 5 outcomes, and 11 quality indicators. Interview data from eight staff members and biometric data from 20 community-dwelling mental health patients taking antipsychotics were evaluated against the framework. Predominantly, patients were managed by the pharmacy’s nurse practitioner, with medication management provided by pharmacists. Patients’ biometric measurements comprised weight, blood pressure, blood glucose levels, lipid profiles and management of obesity, smoking, hypertension and diabetes. Positive outcomes observed in the patient data included weight loss, smoking cessation, and improved blood pressure, blood glucose and lipid levels.

**Conclusions:**

The developed framework allowed effective evaluation of the service, and may be applicable to other pharmacy services. The metabolic clinic met key process, quality and outcomes indicators. The positive patient outcomes may assist in securing further funding.

**Electronic supplementary material:**

The online version of this article (doi:10.1186/s12913-016-1406-6) contains supplementary material, which is available to authorized users.

## Background

Community pharmacists have, over recent decades, positioned themselves to provide disease state management services to supplement patient management by other healthcare providers (e.g. general practitioners). The value of community pharmacy patient-centred disease-state management services has been evaluated in developed countries, with evidence of facilitated patient care and positive patient outcomes [1–4]. Successful funded pilot projects have resulted in service funding by various governments [1–4]. In Australia, community pharmacists are recognised as the most accessible health professionals, and hence are ideally positioned to provide timely healthcare services to patients [1].

The Australian Government has supported trials of disease state management services that promote a role for pharmacists in the management of chronic conditions such as asthma [5], diabetes [6], hypertension [7] and weight management [8], collectively developing a service model for collaborative management of chronic conditions through pharmacies. Whilst these trials reported positive outcomes for patients’ health, commitment by participating pharmacists varied. These trials involved the community pharmacist as the service provider, with referrals to other health professionals as deemed necessary.

No published studies appear to have evaluated the provision of chronic disease management services by other healthcare professionals, such as nurse practitioners (NPs), in community pharmacies. In Australia, a NP is a registered nurse certified to operate independently and cooperatively in a highly developed and extended clinical role. The role of a NP comprises patient assessment and management using nursing knowledge and skills. The role may include, but is not limited to, referral of patients to other health professionals, prescribing of certain government-subsidised medications and ordering of pathology tests [9, 10].

Mental health management services are still under development in Australian community pharmacy practice, despite pharmacists’ skills in monitoring medicine adverse effects and providing clinical interventions [11–13]. Mental health conditions such as anxiety and depression are common, with data showing approximately 50% of the Australian population as likely to experience a diagnosable mental health illness in their lifetime [14]. Management of depression, schizophrenia and bipolar disorder, which may require treatment with antidepressants or atypical antipsychotic agents (e.g. olanzapine, clozapine, risperidone, quetiapine), necessitates a high level of monitoring and multi-disciplinary collaboration. A number of these medications are associated with co-morbidities including weight gain, hyperglycaemia, hypertension and dyslipidaemia [15, 16].

There is a dearth of information about the community pharmacy management of patients taking antipsychotic medicines, with specific reference to the patients’ metabolic risk, and collaboration between pharmacists and NPs. We have attempted to meet this need via evaluation of a novel mental health service developed within a community pharmacy in Perth, Western Australia. One component of this service is a metabolic clinic managed by a full-time NP within the pharmacy. The metabolic clinic was initiated to improve monitoring and manage the metabolic side effects of mental health medications, specifically atypical antipsychotics. This multi-professional service involves in-pharmacy collaboration between the NP and pharmacy staff, as well as external collaboration with patients’ psychiatrists and case managers from a nearby government mental health service. The metabolic clinic is funded by an independent benefactor and underpinned by a Memorandum of Understanding with the government mental health service, to manage up to 300 patients. At the time of the evaluation, 12 months following its initiation, 44 patients had been managed by the metabolic clinic.

Previous evaluation of disease state management services mainly reported patient satisfaction, clinical indicators and quality of life [17–20]. Our evaluation draws on these principles, as well as the integration of ‘good practice’ standards and guidelines, tailored to the service [21]. This service evaluation was endorsed by the pharmacy manager as a quality assurance exercise and an opportunity to reflect on plans for service expansion.

As such, the aim of this project was to conduct a service evaluation of the metabolic clinic for mental health patients. Specific objectives were to:

1. Develop a service evaluation framework to describe the metabolic clinic service, and facilitate analysis and critique of the service.
2. Evaluate the service against the developed framework, and identify areas requiring improvement.

## Methods

Low-risk ethical approval was granted by the Human Research Ethics Committee of Curtin University, Australia (approval number: PH-02-14), for access to pharmacy and de-identified patient data, and for interviews with pharmacy staff. This research was exploratory and involved two stages.

### Stage one: development of a health service evaluation framework

Assessment of the integrative pharmacy-based service required the development of a tailored service evaluation framework. Indicators were compiled from a number of sources involving published literature in the area of quality healthcare, and pharmacy good practice standards and guidelines relating to primary mental healthcare. ‘Process’ indicators were included to evaluate service delivery processes and procedures [17, 22, 23], ‘outcome’ indicators were included to evaluate patient health records [17, 18], and ‘quality’ indicators focused on the significance of care delivered via the service to potentially improve the patients’ health outcomes [20, 24]. All members of the research team concurred on the categorisation of indicators.

### Stage two: evaluation of service elements against the framework

The evaluation comprised two phases to evaluate the existing service against the framework: interviews with the pharmacy staff members, and an evaluation of the metabolic clinic patients’ records.

#### Staff interviews

Semi-structured qualitative interviews were conducted with pharmacy staff during March 2014, to obtain insight into the running of the metabolic clinic to facilitate analysis of the service procedures and staffing contributions. Interviews were identified as a more practical solution for engaging with individual staff members with different work rosters compared to a focus group(s); this method also allowed tailoring of questions to staff members’ roles in the metabolic clinic. All staff with some involvement with the metabolic clinic, and who worked during the operational hours of the metabolic clinic service, were included. Two interview guides were developed by the research team and validated by the pharmacy owner, who ensured relevance of the questions; suggested changes were incorporated. The interview guide for the pharmacists (Additional file 1) and (Additional file 2) NP comprised 10 questions focusing on the processes and operation of the metabolic clinic service, the role of staff members and potential improvements to the service (i.e. process and quality indicators). The questions were slightly modified for pharmacy assistants to reflect their scope of practice. The use of open-ended questions and prompts encouraged staff to detail their experiences and explore ideas, and generated subjective and objective data. Interviews were conducted within the pharmacy premise, with signed consent for audio recording.

Recordings were transcribed and annotated electronically by the primary researcher, with data categorised under the relevant process or quality indicator. Content analysis was confirmed by the collaborating researchers, and data were reported descriptively, supported by de-identified quotations.

#### Patient data collection

A spreadsheet of de-identified patient biometric information was provided by the NP to provide insight into outcome indicators. The spreadsheet was evaluated for the main parameters that the NP recorded: weight, height, body mass index, waist circumference, blood pressure and blood glucose levels, lipid profile, heart rate, respiratory rate and oxygen saturation. Missing data were annotated using verbal explanations by the NP. In light of the recent commencement of the service and number of patients enrolled, specific patient cases, based on positive outcomes achieved, were reported as case studies in lieu of statistical analysis of within- and inter-patient outcomes data, and to demonstrate the breadth of the service.

## Results

### Stage one: Development of the health service evaluation framework

The evaluation framework (Table 1) comprised 13 process indicators, five outcome indicators and 11 quality indicators.

**Table 1:** Metabolic risk health service evaluation framework

Process Indicators [17, 22, 23]	Outcome Indicators [17, 18]	Quality Indicators [20, 21, 24]
• Patient initiation with the metabolic clinic i.e. through the government mental health service • Enrolment process • Case load, i.e. how many patients can be managed, workload implications, time per consultation • Management of patients’ issues at the lowest appropriate level (i.e. lowest primary health care level and minimum level of service) • Service components provided by nurse practitioner *vs* pharmacist *vs* pharmacy assistant • Process involved in outcomes reporting [17] • Referrals and associated communication [23] • Follow-up of the patient [23] • Consultation fee (eligibility for funded services *vs* fee-for service) • Data recorded at different points of consultation [22] • Baseline data collection [22] • Mode of documentation [23] • Monitoring of patient improvement and evaluation of progression [21]	• Qualitative data (free-text notes, reflections) [18] • Patient compliance with medication, relative to adverse effects experienced, pill counting and dispensing or supply intervals [18] • Improvement in overall health status of patient (physical and mental health) • Data collected (e.g. biometric parameters, subjective assessments, patients’ concerns) [17] • Review of the data/readings obtained [18]	• Patient privacy and confidentiality [21] • Treatment plan customisation for each patient [20] • Level of patient assessment. Is it only based on improving physical symptoms (confirmed by lab tests), or does it also include psychological and emotional improvement? [20, 24] • Patient education regarding their condition, treatment and medication side effects [20] • Simplicity of information (verbal and written) provided to patients (review sources of information) [20] • Up-to-date record keeping [20] • Level of pharmacists’ communication with other health professionals [20, 21] • Staff understanding that mental health patients may feel stigmatised and hence treat patients in an understanding manner (staff completing training specific to mental health) [20] • Pharmacist involvement in professional development courses to enhance knowledge in providing the disease state management service [21] • Promotional activities undertaken in relation to mental health, as a form of encouragement for current patients [20] • Improvements in the metabolic clinic service

### Stage two: evaluation of service elements against the framework

From the pharmacy’s 12 staff members, 10 met the inclusion criteria and consented to participate in an interview. The two excluded staff members were not employed during the operational hours of the metabolic clinic. Participants comprised six pharmacists, one NP and three pharmacy assistants. Interviews ranged from 20 to 40 min each (mean 30 min).

The following sections report the process, outcome and quality indicators relating to the metabolic clinic service. Process and quality data were derived from the interviews, while outcome data were derived from the patients’ biometric data. No additional categories were identified for inclusion in the evaluation framework during the analysis. Paucity of patient outcomes data meant a number of outcomes indicators were unable to be assessed.

#### Process indicators

Data provided by pharmacy staff directly addressed six of the 12 process indicators (Table 2). The pharmacy manager described the target patient group as patients prescribed atypical antipsychotics; these patients received the metabolic clinic service free of charge, as per the funding arrangement. Non-government mental health patients were required to make a payment of AUD$25 per consultation.

**Table 2:** Process indicators

Process Indicators	Examples
Patient initiation with the metabolic clinic i.e. through the government mental health clinic	Predominantly patients on atypical antipsychotics
Enrolment process	No official paper work existed in initiating patients into the metabolic clinic service
Consultation fee (eligibility for funded services *vs* fee-for service)	Mental health patients on atypical antipsychotics received the service free of charge as per the funding model
Referrals and associated communication	Patients were referred to the Metabolic Clinic Service via staff at the government mental health service (psychiatrists, case managers)
Service components provided by nurse practitioner *vs* pharmacist *vs* pharmacy assistant	Proprietor oversaw the operation of the metabolic clinic. The pharmacist who was next in charge was involved in promotional activities at the government mental health clinic. All staff were actively involved in referring patients to the metabolic clinic
Mode of documentation	Patient biometrics were recorded in an Excel spreadsheet. The NP used the Good Practice® software to record information relating to consultations. Changes relating to medication and/or dosage were handwritten in patient specific files

The metabolic clinic was overseen by the pharmacy manager, who monitored patient progress, ensured the smooth running of the service and evaluated six-monthly reports received from the NP. One of the pharmacists conducted promotional presentations, mainly at the government mental health service and a residential care facility, to ensure health professionals and patients were aware of the clinic and its relevance to patients’ health. All pharmacy staff were involved in referring mental health patients they considered at risk of adverse medication effects to the clinic.

Staff members were of the opinion that official paperwork was a barrier to patient management. Initially, the pharmacy provided referral forms to be completed by the staff at the government mental health service to introduce patients to the pharmacy service, but these did not assist in the pharmacy workflow.

Any adjustments to mental health patients’ therapy by the staff at the government mental health clinic, identified during dispensing or other communications with prescribers, were annotated by pharmacists in patient-specific files kept in a designated area within the dispensary. Changes to the patients’ medications were reportedly common and were recorded manually in the files and in the dispensing software at the time of dispensing, so all pharmacists were able to view changes. The NP recorded patient biometric data in a spreadsheet, and other consultation information in the Best Practice® software kept in the metabolic clinic.

#### Quality indicators

Interview data addressed six of the 11 quality indicators (Table 3). To uphold privacy and confidentiality requirements, patients were required to sign a consent form when initiating with the metabolic clinic. In terms of data security, the information recorded in the Best Practice® software was password protected. With the permission of the NP, the pharmacy manager was, however, able to access patient information in the event of an emergency.

**Table 3:** Quality indicators

Quality Indicators	Examples
Patient privacy and confidentiality	Patients were required to sign a consent form before signing up with the metabolic clinic service
The level of patient assessment. Is it only based on improving physical symptoms (confirmed by lab tests), or does it also include psychological and emotional improvement?	Metabolic clinic assessments were based on physical parameters, such as blood pressure, blood glucose levels, lipid profile and weight
Patient education regarding their condition, treatment and medication side effects	Staff addressed medication compliance issues, by educating the patient on the importance of his/her medication and its relevance to his/her health
Pharmacist involvement in professional development courses to enhance knowledge in providing the disease state management service	Pharmacists were actively involved in professional development courses, such as Mental Health First Aid
The level of pharmacists’ communication with other health professionals	Pharmacists were in regular contact with psychiatrists and staff members at the government mental health clinic
Improvements to the metabolic clinic service	The study pharmacy required further promotion of its service for greater patient numbers.
	Integration between the NP’s software and pharmacists dispensing software was required for up-to-date patient medication data

Patient assessment was based on biometric parameters. Generally, the first set of blood tests requested common haematological parameters, and depending on the results, the NP followed up with more specific pathology orders. A vitamin D test was routinely ordered for all patients, and depending on initial screening, iron levels would be tested, regardless of the patient’s gender. Age-appropriate tests were also ordered; for example, an overweight male over 50 years of age would also undergo a Prostate Specific Antigen test.

Patients’ mental health status was assessed through various communication and observation strategies, with staff members noting their emotional states and whether the patient appeared to comprehend information provided by staff. Pharmacists managed issues within their scope and confidence level, and referred patients to relevant professionals. Overall, the pharmacists viewed their communication with other health professionals, with specific relevance to the staff at the government mental health service, as effective and productive. Pharmacy staff used the mobile telephone numbers of the psychiatrists and case managers in emergencies, while other modes of contact were used to discuss less-urgent patient matters. One of the pharmacists was also a liaison pharmacist with the government mental health service, and was in frequent contact with the facility. Patient education facilitated medication adherence, and staff adopted a holistic approach by also addressing the importance of healthy lifestyles and monitoring.

One area for improvement identified was a need for promotion of the metabolic clinic at other mental health clinics within close proximity of the pharmacy, to increase health professional and patient awareness. A second shortcoming was the lack of communication between the pharmacists’ dispensing software and the NP’s Best Practice® software, with the latter software the same as that utilised by many prescribers. When the NP’s medication records were not current, the NP checked the dispensing software for recent changes. This was identified as not only inconvenient, but potentially compromising patient care. The pharmacy was in the process of implementing new software to integrate the dispensing and the NP’s systems. Furthermore, the handwritten annotations regarding patient progress by pharmacists’ in patient files may increase the likelihood of human error. Handwriting was recognised as a risk for misinterpretation of medication names, dosages and frequencies. Hard-copy patient files may also be misplaced in a busy work environment.

#### Outcome indicators

The patient spreadsheet contained biometric information for 20 patients for the period November 2013 (commencement of the current service contract) to March 2014 (close of data collection). Data for 24 patients were not provided, in accordance with privacy requirements under a previous service agreement. Positive trends were noted for the 20 patients, although the number of follow-up visits to the clinic (and hence, data) varied between patients. Improvements in physical health risk indicators were observed for most patients during the operation of the clinic.

Three patient cases illustrative of those with clinically-significant outcomes over the short follow-up period are presented in Table 4.

**Table 4:** Patient cases

Weight reduction, improved blood pressure control	Improved lipid profile	Increased physical activity and smoking cessation
Mrs X: 30 years old	Mr Y: 42 years old	Mr Z: 71 years old
Data were available from the patients’ second through sixth appointment. During this period, the patient was able to implement an effective diet and exercise program with the assistance of the NP, which allowed her to lose weight and reduce her blood pressure. She lost six kilograms, and her blood pressure reduced from 131/88 to 125/75. Her waist circumference reduced from 133 cm to 120 cm. While she had not incorporated extensive changes to her lifestyle, simply being able to make small adjustments in her habits has enabled improvements. These small changes include walking her dog at a faster rate, stepping up and down a curb and making better choices for breakfast, such as smoothies. Whilst she may still opt for something sugary, it is now a matter of eating a small bar of chocolate rather than a bag of lollies. This case portrays an ideal case of a patient who has been able to incorporate small changes, and achieve results. She visited the pharmacy frequently and consulted with the NP. This indicates the significance of the metabolic clinic, as well as the patient-centred service provided by the pharmacy.	Mr Y was identified as being overweight, requiring a diet and exercise program. A blood test result revealed high lipid levels, with total cholesterol being 7.9 mmol/L and triglycerides being 5.7 mmol/L. As a result, the NP initiated him on rosuvastatin (Crestor®). This medication reduced his total cholesterol to 5 mmol/L and triglycerides to 2.8 mmol/L. Six months later, the patient decided to cease the rosuvastatin (Crestor®), as he did not understand why there was a need for him to continue. Following this, another blood test result showed raised levels, with total cholesterol being 8.6 mmol/L and triglycerides 6.2 mmol/L. After the patient was shown these results, he re-commenced rosuvastatin (Crestor®). It was hoped that he would return for future consultations and blood tests. In this case, the NP was able to educate the patient on the importance of his medication, by explaining his test results and reasoning why those values had increased, which proved to be beneficial advice for the patient.	After being admitted to hospital, possibly with chest pain or a myocardial infarction, Mr Z was encouraged to change his lifestyle, with the assistance of the NP. In a period of two months, his systolic blood pressure had risen from 140/100 to 153/78. In the space of one month, effective lifestyle changes allowed for his blood pressure to reduce to 112/54. The NP described him as an extremely motivated individual; he would come into the pharmacy more frequently than his hospital visits. The major change he incorporated into his life was increasing his physical activity by cycling. After his emergency visit to the pharmacy, he also undertook smoking cessation with the support of the NP.

## Discussion

The novel pharmacy service evaluated was established in 2013, and was still undergoing development at the time of the study. The service was introduced in the absence of an evaluation framework; as such, this independent review of systems and processes was timely for reflection and quality improvement.

The evaluation of this metabolic clinic service, which represents a unique model of collaboration between a NP and pharmacists, suggests potential for the development of other multi-disciplinary health services in community pharmacies. The metabolic clinic was predominantly operated and managed by the NP, with pharmacists mainly involved in routine medication management and referral of patients to the service. In Australia, registered NPs have formalised roles allowing them to prescribe certain government-remunerated medication, order pathology tests and administer injections, thereby enabling NPs to manage patients in collaboration with other health professionals [25]. The pharmacists played a significant role in facilitating patient health literacy and educating them about their medication in order to improve adherence, complementing the NP’s role. This multi-professional approach allowed both the NP and pharmacists to provide patient care in their own fields of expertise, using a holistic and collaborative model of care.

Australian trials over the past decade have revealed positive patient outcomes in community pharmacist-led management of asthma [5], diabetes [6] and hypertension [7]. Common features of these multi-site studies were the leadership of pharmacists in developing a therapeutic relationship with patients at risk of complications, collaborative identification of therapeutic goals, and provision of counselling and referrals to assist patients in achieving these goals. In none of these studies was a NP (or any other non-pharmacist health professional) employed in the pharmacy to collaborate in patient management. This collaboration, drawing on the unique scope of practice of NPs, is proposed to further enhance ambulatory management of patients with chronic conditions. While it was not feasible to attribute ‘cause and effect’ of the NP’s interventions in the three cases presented, the patients’ progress and commitment suggest impact of the NP’s role over a relatively short period.

The pharmacists’ role in referral to the metabolic clinic and routine medication management complemented the role of the NP. Pharmacy assistants were primarily involved in referring ‘at-risk’ patients to the pharmacists for consultation. The NP took responsibility for the majority of patient management, documentation and referrals. There were no time restrictions on the NP to complete consultations, which facilitated patient-centred and quality care. Collectively, this teamwork appeared effective, and with an appropriate service contract, could be applied to other settings and health conditions. Areas for improvements highlighted by the evaluation – the need for integrated software and promotion of the service – were already recognized by the participants.

There were limitations in our method of evaluation. This study only evaluated patient data over a five-month period, as previous data were unavailable for analysis. In addition, the study did not incorporate economic evaluation, due to the confidentiality of business data. However, our early-stage evaluation has been adopted as part of the pharmacy’s culture of continuous quality improvement, which may include economic evaluation. The funding arrangement with the independent benefactor ensured financial viability for 1000 patients and relieved the pharmacy manager of financial pressure and concerns about financial viability of the metabolic clinic.

In relation to the interview process, reporting bias from the staff members could not be discounted. All staff involved in the service, regardless of their role in instigating or otherwise providing the service, were included and were encouraged to critically reflect on its operation. As with all qualitative research, the background and perspectives of the researcher may have an impact on analysis of the data. However, all members of the research team were involved in the analysis presented here.

We now offer this evaluation framework as a starting point for evaluation of other patient-focused services, acknowledging that the number of criteria could be reduced or adapted to other settings, and resource indicators could be added to reflect physical requirements for service establishment and provision. The study pharmacy was deemed to be providing an overall effective service in relation to all three indicators, despite the interview data mapping to only a portion of the constituent criteria.

## Conclusions

This service evaluation met its objectives of developing an evaluation framework that may be refined and applied to patient-focused health management services. The subsequent evaluation identified that the metabolic clinic service was meeting key indicators including process, outcomes and quality indicators, and the positive patient outcomes may facilitate further funding from the independent benefactor. Areas requiring improvement, namely electronic data management and increased promotion, were identified. Awareness of these limitations and practical solutions will ensure the service is positioned for regular evaluation and allow for expansion.

## Availability of data and materials

The raw data are not published in order to protect the identity of participants. Requests for de-identified raw data will be considered by the authors.

## Additional files

Additional file 1: Semi-structured interview guide (Nurse Practitioner and Pharmacists). (DOCX 37 kb) Additional file 2: Semi-structured interview guide (Pharmacy Assistants). (DOCX 37 kb)
