# Supplementary material for: Can a pharmacy intervention improve the metabolic risks of mental health patients? Evaluation of a novel collaborative service
Source: BMC Health Serv Res. 2016 Apr 26;16:146. doi: 10.1186/s12913-016-1406-6 (PMC4845305; doi:10.1186/s12913-016-1406-6)
Supplement: Additional file 1: — Semi-structured interview guide (Nurse Practitioner and Pharmacists). (DOCX 37 kb) [file 12913_2016_1406_MOESM1_ESM.docx]

#
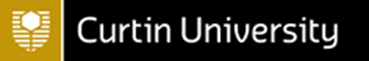
School of Health Science

# Bentley Campus, Curtin University

Kent Street, Bentley, Perth

Western Australia. 6102.

[15564766@student.curtin.edu.au](mailto:15564766@student.curtin.edu.au)

Semi-structured interview guide

### [Name deleted] Pharmacy Staff Members

### (Nurse Practitioner and Pharmacists)

**Can a Pharmacy Intervention Improve the Metabolic Risk**

**of Patients taking Mental Health Medicines?**

The semi structured qualitative interviews with [name deleted] Pharmacy staff members will focus on:

1. Developing a service evaluation framework suitable for analysis and critique of the Metabolic Clinic service
2. Evaluating the service against the developed framework and identify areas requiring improvement
3. Identifying either an intervention to be trialled, or a time point at which an intervention was introduced, in order to monitor the effects of this intervention

Give a brief introduction.

Show gratitude for participation effort.

Ensure consent form has been read, signed and is returned.

Turn on the recorder.

Explain the need for the project:

We are interested in conducting a service evaluation of the Metabolic Clinic service recently developed by [name deleted] Pharmacy.

So before we get into thorough details about your experiences with the Metabolic Clinic, I just want to begin with asking you about your history.

1. How have you been involved in the pharmacy?

- Describe your role within the pharmacy, and whether it is part-time or full-time.
- Describe your qualifications and your experience within pharmacy.
- Approximately how many patients have you identified and referred to the Metabolic Clinic?

2. Describe your role within the Metabolic Clinic.

- What is the difference between your role and that of the nurse practitioner/pharmacist (as appropriate)?
- Do the staff have pre-determined roles within the Metabolic Clinic?

3. Why do you feel there was a need for a Metabolic Clinic within a pharmacy setting?

- Throughout your experience, what have been the outcomes from the Metabolic Clinic?

Prompts:

- Patient (improved compliance), pharmacist (improved inter-professional relationships with GPs)
- Can you think of any particular Metabolic Clinic patients who have made significant progress? I will be evaluating their files, but am interested in memorable success stories from the staff’s perspective.
  - Who contributed to this success?
- Are mental health patients on atypical antipsychotic therapy the main target of the Clinic, or does the service extend to the public as well?
- Does the Metabolic Clinic service extend to all mental health patients, such as those on SSRIs or other antidepressants, or is it specifically for patients on atypical antipsychotics?
- How do you feel about the targeting of people who can potentially benefit from the Metabolic Clinic service (at-risk groups)?

Prompt:

- Whether high risk groups (adolescents and/or elderly mental health, patients without support structures) have been efficiently targeted

4. As a pharmacist/nurse practitioner, describe the process you utilise in identifying whether a patient is likely to benefit from the Metabolic Clinic.

- Is there a strict protocol to be followed, or is the service tailored on a case-by-case basis?

5. How aware are other health practitioners in the community about the [name deleted] Pharmacy Metabolic Clinic service?

- Are referrals mainly from the [name deleted] Mental Health Service or elsewhere?

6. In relation to record keeping and type of data collected from patients, is there a specific protocol that must be followed?

- Are there limitations as to the patient data that can be accessed by different staff members?
- Are there guidelines for patient follow-up?

7. What aspects do you think are working well for this Metabolic Clinic service?

8. What are your suggestions to improve the Metabolic Clinic in specific to promote uptake, effectiveness and usefulness?

- Which one of these suggestions do you think would be the best?

9. What sort of factors do you feel may influence the extent of success of a Metabolic Clinic?

Prompts:

- Location of the pharmacy in close proximity to major hospitals
- Pharmacy set-up: private consultation areas
- Having a nurse practitioner and a pharmacist working collaboratively with other health professionals (GP, psychiatrist)
- Payment for the service – patients paying versus a sponsored program
- Can you describe some ways that you think GPs and pharmacists could work more efficiently together in a collaborative team approach to achieve a better outcome?
- (if had patients who refused the Metabolic Clinic service) Can you name some common reasons stated by patients?

10. (Manager) Would you please explain the funding of the Metabolic Clinic service and government remuneration involved?

11. Are there any more comments you would like to make regarding your experience with the Metabolic Clinic service that you would like to share?

Thank you so much for your time and effort.
