# Supplementary material for: Can a pharmacy intervention improve the metabolic risks of mental health patients? Evaluation of a novel collaborative service
Source: BMC Health Serv Res. 2016 Apr 26;16:146. doi: 10.1186/s12913-016-1406-6 (PMC4845305; doi:10.1186/s12913-016-1406-6)
Supplement: Additional file 2: — Semi-structured interview guide (Pharmacy Assistants). (DOCX 37 kb) [file 12913_2016_1406_MOESM2_ESM.docx]

#
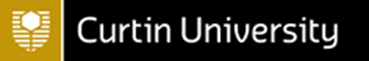
School of Health Sciences

# Bentley Campus, Curtin University

Kent Street, Bentley, Perth

Western Australia. 6102.

[15564766@student.curtin.edu.au](mailto:15564766@student.curtin.edu.au)

Semi-structured interview guide

### [Name deleted] Pharmacy Staff Members

### (Pharmacy Assistants)

**Can a Pharmacy Intervention Improve the Metabolic Risk**

**of Patients taking Mental Health Medicines?**

The semi structured qualitative interviews with [name deleted] Pharmacy staff members will focus on-

1. Developing a service evaluation framework suitable for analysis and critique of the Metabolic Clinic service
2. Evaluating the service against the developed framework and identify areas requiring improvement
3. Identifying either an intervention to be trialled, or a time point at which an intervention was introduced, in order to monitor the effects of this intervention

Give a brief introduction.

Show gratitude for participation effort.

Ensure consent form has been read, signed and is returned.

Turn on recorder.

Explain the need for the project:

We are interested in conducting a service evaluation of the Metabolic Clinic service recently developed by [name deleted] Pharmacy.

So before we get into thorough details about your experiences with the Metabolic Clinic, I just want to begin with asking you about your history.

1. How have you been involved in the pharmacy?

- Describe your role in the pharmacy and whether it is part-time or full-time.
- Describe any qualifications you may have and your experience held within the pharmacy.

2. In your position, can you refer patients to the Metabolic Clinic?

- (If yes) How many patients have you identified and referred to the Metabolic Clinic?
- Have you had any patients decline your offer of the service? If yes, can you state some of the common reasons?

3. Do the staff have pre-determined roles in relation to the Metabolic Clinic?

- Specifically, how are you involved?
- Is there a strict protocol to be followed, or is the service tailored on a case-by-case basis?
- What is the level of involvement of pharmacy assistants compared to the nurse practitioner and pharmacist in relation to the Metabolic Clinic?

Prompt:

- Do you simply refer patients to the nurse practitioner and/or pharmacist or is there a questioning process beforehand? (For e.g. how would you identify someone for this service?)
- Are there limitations as to the patient data that can be accessed by different staff members?

4. Throughout your experience, what outcomes have you seen from the Metabolic Clinic? Prompts:

- Patients’ physical/mental health (e.g. improved medication/treatment compliance), pharmacist (improved inter-professional relationships with GPs)
- Can you think of any particular patients who have made significant progress? I will be going through their files, but am interested in memorable success stories from the staff’s perspective.
  - Who contributed to this success?
- Are mental health patients on medication the main target of the Clinic, or does the service extend to the public as well?
- Does the Metabolic Clinic service extend to all mental health patients, or is it specifically for patients on certain types of medication?
- How do you feel about the targeting of people who can potentially benefit from the Metabolic Clinic service (at-risk groups)?

Prompt:

- Whether high-risk groups (adolescents and/or elderly mental health, patients without support structures) have been efficiently targeted?

5. What aspects do you think are working well for this Metabolic Clinic service?

Prompt:

- Contribution of a nurse practitioner within the pharmacy and involvement of all pharmacy staff?

6. What are your suggestions to improve the Metabolic Clinic to maximise its uptake, effectiveness and usefulness?

- - Which one of these suggestions do you think would be the best?
  - Describe ways in which more health professionals could be encouraged to get more involved in the program.

7. Do you believe that training is required for the pharmacy assistants involved in assisting in the Metabolic Clinic service?

Prompt:

- Enhancing education in mental health conditions and/or medication
- Enhancing communication skills

8. What sort of factors do you feel may influence the extent/success of the Metabolic Clinic?

Prompts:

- Location of the pharmacy in close proximity to major hospitals
- Having a nurse practitioner and a pharmacist working collaboratively with other health professionals (GP, psychiatrist)
- Payment for the service – patients paying versus a sponsored program
- Can you describe some ways that you think GPs and pharmacists could work more efficiently together to achieve a better outcome?

9. Are there any more comments you would like to make regarding your experience with the Metabolic Clinic service that you would like to share?

Thank you so much for your time and effort.
